# Fistules carotido-caverneuses bilatérales spontanées: à propos d’un cas et revue de la littérature

**DOI:** 10.11604/pamj.2017.27.91.8594

**Published:** 2017-06-06

**Authors:** Adil Belhachmi

**Affiliations:** 1 Service de Neurochirurgie et Neuroradiologie Interventionnelle, Hôpital Chekh Zaid Hay Riad, Rabat, Maroc; 2 Service de Neurochirurgie, Hôpital Militaire, Rabat, Maroc

**Keywords:** Exophtalmies bilaterales, fistule, embolisation, Bilateral exophtalmos, fistula, embolization

## Abstract

Nous rapportons une observation clinique d'une fistule carotidocaverneuse survenue spontanée avec Une exophtalmie biilatérale pulsatile et symétrie des 2 sinus caverneux au scanner ont permis de suspecter le diagnostic. Une artériographie a permis de confirmer ce diagnostic, avec embolisation couronnée de succès chez cette malade, avec une évolution est favorable sur le plan neurologique et ophtalmique. La fistule carotido-caverneuse est une complication rare mais grave pouvant engager le pronostic fonctionnel (cécité) et vital (hémorragie méningée et intracérébrale). L'artériographie et l'embolisation en un seul temps ont considérablement amélioré le pronostic.

## Abstract (en)

We report a clinical case of spontaneous bilateral carotid-cavernous fistulas. Bilateral pulsatile exophthalmos and symmetric cavernous sinuses detected by CT scan helped to suspect the diagnosis. Arteriography confirmed the diagnosis. The patient underwent embolization with favorable neurologic and ophthalmic evolution. Carotid-cavernous fistula is a rare but severe complication associated with a poor functional (blindness) and vital (meningeal and intracerebral hemorrhage) prognosis. The concomitant use of arteriography and embolization has considerably improved the prognosis.

## Introduction

Les fistules carotidocaverneuses spontannée sont des communications anormales entre le système carotidien et le sinus caverneux,. C'est une complication rare, et fres exceptionnelle dont le diagnostic est suspecté en clinique. La situation profonde du sinus caverneux rend le traitement chirurgical difficile. Le pronostic s'est largement amélioré ces 20 dernières années grâce aux progrès de la neuroradiologie interventionnelle. Le but de ce travail est de présenter les premiers signes cliniques et para cliniques qui peuvent orienter ver le diagnostic de FCC, l'intérêt de la rapidité et l'efficacité du traitement endovasculaire et leurs impacts sur le pronostic vitale.

## Patient et observation

**Figure 1:**
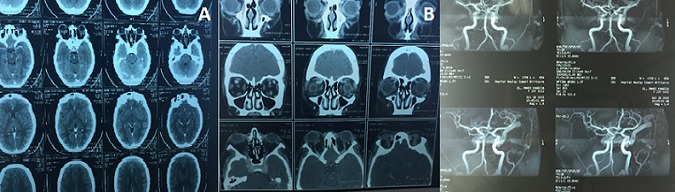
(A) TDM cérébrale montre une hémorragie méningée, asymétrie des 2 sinus caverneux; (B) IRM cérébrale injectée gado: montrant au temps artériel des fistules carotido-caverneuse bilatérale, avec opacification précoce des 02 sinus caverneux

**Figure 2:**
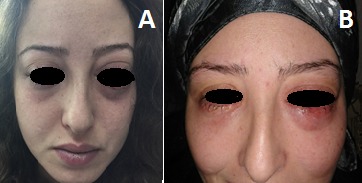
(A) artériographie carotidienne montrant les 02 fistules carotido-caverneuse bilaterale (avant); (B) artériographie carotidienne montrant les 02 fistules carotido-caverneuse (après embolisation)

**Figure 3:**
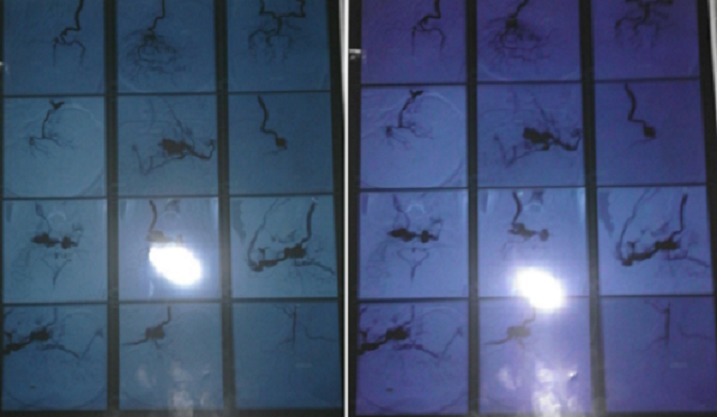
Artériographie post embolisation

Une jeune femme de 22 ans, sans antécédent, qui constate depuis 04 mois des céphalées chroniques et une exophtalmie bilatérales progressives des 02 yeux, patient admis au service d'ophtalmologie, dont le médecin a demandé une IRM cérébrale puis adressée à notre service pour prise en charge d'une fistule carotido caverneuse bilaterale. Le scanner cérébrale trouve une hémorragie méningée avec asymétrie des 2 sinus caverneux. Ces signes ont fait douter une FCC, une artériographie (Figure 1 A, Figure 1 B) et une embolisation a été réalisé avec exclusion de FCC par des ballonnets largables, avec une bonne perfusion des 02 carotides internes le contrôle angiographique (Figure 2 A, Figure 2 B, Figure 3) a objectivé une occlusion complète de la fistule avec une bonne perfusion de la carotide interne perméable et une bonne circulation au niveau du polygone de Willis. L'évolution favorable sur le plan neurologique et ophtalmique.

## Discussion

La fistule carotidocaverneuse est un shunt artério-veineux anormale entre le système carotidien et le sinus carverneux. Elle est le plus souvent unilatérale, cependant quelques cas de formes bilatérales ont été décrites. Dans les suites d'un traumatisme crânien, on observe surtout les fistules artérioveineuses directes à débit élevé, type A de la classification de Barrow [1]. Classification angiographique des fistules carotidocaverneuse selon Barrow et al. 1985. Type A: shunt direct entre l'ACI et le sinus caverneux; type B: shunt dural entre les branches méningées de l'ACI et le sinus caverneux; type C: shunt dural entre les branches méningées de l'ACE et le sinus caverneux; type D: shunt dural entre les branches méningées provenant à la fois de l'ACE et de l'ACI et le sinus caverneux. ACI: artère carotide interne; ACE: artère carotide externe. La fistule entraine une communication anormale du flux artériel vers le sinus caverneux et ses affluents.ces structures sont inappropriées pour contenir un sang circulant à haut débit et à haut pression. On observe une dilatation du réseau veineux d'amont avec artérialisation [2]. Les signes cliniques oculaire sont prépondérants du fait de la position d'amont des veines ophtalmiques par rapport au sinus caverneux. Une exophtalmie pulsatile est retrouvée dans 90% des cas, associé à une baisse de l'acuité visuelle dans 80% des cas (Mais parfois l'exophtalmie est minime et non pulsatile, difficilement trouvable à l'examen clinique) [3]. L'auscultation de la région périorbitaire et temporale retrouve un souffle intracrânien systolo-diastolique disparaissant à la compression manuelle de l'artère carotide homolatéral au niveau du cou (Cette symptomatologie peut être absente ou bien méconnue dans les grands fracas cranio-faciaux avec oedème important du visage et la FCC sera révélée plusieurs mois, voire plusieurs années après le traumatisme par des céphalées, des manifestations ophtalmologiques ou bien par une hémorragie cérébrale ou sous arachnoïdienne) [4].

L'exploration neuroradiologique constitue un temps essentiel dans le diagnostic et le traitement des FCC post-traumatiques. L'écho-doppler couleur permet d'affirmer la fistule en montrant au niveau des veines ophtalmiques un signal doppler inversé dirigé vers la face à renforcement systolique. Cet permet en outre un suivi après embolisation ou abstention thérapeutique. Le doppler transcranien visualise directement la fistule avec une sensibilité de 95% (mais il est opérateur dépendants). La tomodensitométrie cérébrale recherche le plus souvent les signes indirects, qui sont ipsilatéraux à la fistule ou parfois bilatéraux : élargissement du sinus caverneux et de la veine ophtalmique supérieure, infiltration des muscles oculomoteurs et des tissus orbitaires. L'artériographie cérébrale est l'examen de certitude de FCC, et surtout mise en oeuvre des thérapeutiques dans le même temps interventionnel. Hmamouchi et al.préconisent même sa réalisation d'emblée devant une exophtalmie pulsatile associée à un souffle orbitaire systolo-diastolique [5]. L'évolution est marquée par troubles ophtalmologique et neurologique, sur le plan neurologique, il a été observé des complications secondaires aux FCC à partir d'une série de 155 patients [6]. Les plus fréquentes sont la varice du sinus caverneux, le drainage veineux cortical non physiologique, potentiellement responsable d'une hypertension intracrânienne et d'hémorragie intracrânienne, le pseudo anévrysme post-traumatique et l'épistaxis massive. Enfin, une résolution spontanée de ces FCC est démontrée dans 5 à 10% des cas dans une série de 132 patients, dans notre étude après le traitement de FCC [6] (le patient a présenté une hypertension intracrânien sévère réfractaire au traitement médicale, probablement en rapport avec son état neurologique initial défavorable (GCS à 6) aggravé par l'embolisation précoce; donc faut-t-il traiter précocement les patients présentant des FCC post traumatiques ou préféré un traitement tardif après stabilisation de l'état clinique du patient.

## Conclusion

La FCC spontanée est une complication très rare mais grave pouvant engager le pronostic fonctionnel ou vital, leur diagnostic clinique est évoqué sur des signes ophtalmiques et orbitaires qui sont à rechercher activement. La surveillance quotidienne par le doppler transuranien peut permettre leur dépistage précoce et indiquer des explorations complémentaires. La visualisation des signes directs ou indirects sur le scanner cérébrale doit conduire à réaliser une angiographie cérébrale diagnostique et thérapeutique. La place de la neuroradiologie interventionnelle semble indiscutable en traitement de première ligne. Les indications de sacrifice chirurgical de la carotide se résument actuellement aux échecs du technique endovasculaire.

## Conflits d’intérêts

Les auteurs ne déclarent aucun conflit d'intérêts.
